# Ultrastructural study on the granulocytes of Uttara fowl (*Gallus domesticus*)

**DOI:** 10.14202/vetworld.2016.320-325

**Published:** 2016-03-26

**Authors:** Khan Idrees Mohd, Meena Mrigesh, Balwinder Singh, Ishwar Singh

**Affiliations:** Department of Veterinary Anatomy, College of Veterinary & Animal Sciences, G.B. Pant University of Agriculture and Technology, Pantnagar, Uttarakhand, India

**Keywords:** blood cells, cytoplasmic granules, ultrastructure, Uttara fowl

## Abstract

**Aim:**

The present study was conducted to know the ultrastructural detail of the blood cells of Uttara fowl (native fowl of Uttarakhand).

**Materials and Methods:**

The experiment was conducted on 10 apparently healthy adult birds of either sex reared at the Instructional Poultry Farm, G.B. Pant University of Agriculture and Technology, Pantnagar, Uttarakhand. The blood was collected from wing vein using ethylenediamine tetraacetic acid as anticoagulant. The blood was further processed for scanning and transmission electron microscopic (TEM) studies separately.

**Results:**

Ultrastructurally, the heterophils were irregularly round in shape. The cytoplasm was laden with pleomorphic membrane-bound granules, *viz*., large elliptical-, medium oval-, large round-, and medium round-shaped granules. The eosinophils under TEM were irregularly circular in outline showing pseudopodia and finger-like cytoplasmic processes. The cytoplasmic granules were pleomorphic with elliptical-, round-, and rod-shaped granules. The basophils were irregularly circular in outline containing small hook-like cytoplasmic processes. The cytoplasm contained electron dense and electron lucent round-shaped granules.

**Conclusion:**

Granulocytes contained pleomorphic cytoplasmic granules. However, the shape and electron density of granules varied among the different granulocytes and helped in the characterization of different granulocytes.

## Introduction

India has emerged as the third largest egg producer and fifth largest poultry meat producer in the world. The total chicken population has registered an annual growth of 7.3% in the last decade. Farm chicken grows at 12.4%, whereas Desi chicken showed much lower growth rate of about 2% [1]. Backyard poultry farming has over the years contributed to a great extent to the agrarian economy of India. An important part of the poultry meat and eggs consumed in the country comes from these small-scale producers. Backyard poultry farming offers a great scope and has immense potentials in hills of Uttarakhand. The indigenous hill fowl is the backbone of the backyard poultry farming in hills. The Uttara fowl (local hill fowl) is said to be descended from the Red Jungle fowl. Most of the hill fowls are unique in their adaptation to the agro-climatic conditions of their habitat [2].

The mammalian analog of neutrophil in the bird is called heterophil. These are the first line of defense in the body and play an indispensable role in the immune defense of the avian host. To accomplish this defense, heterophils use sophisticated mechanisms such as phagocytosis, degranulation, and oxidative burst to destroy pathogenic microbes [3]. Their cytoplasmic granules contain several lysosomal and non-lysosomal enzymes including acid phosphatase, arylsulfatase, β-glucuronidase, phosphorylase, neutral and acid α-glucosidases, acid trimetaphosphatase, and lysozyme [4].

Keeping in view, the use of granules in the defense function of the cells, the present study was undertaken to study the general ultrastructural details of the granules present in different granulocytes. Although the reports on granulocyte ultrastructure in other birds such as guinea fowl, ostrich, and painted stork are available. However, the reports on ultrastructural features of granulocyte cells in Uttara fowl are not available. Keeping it in view the above facts, the present study was conducted using scanning electron microscope (SEM) and transmission electron microscope (TEM).

## Materials and Methods

### Ethical approval

The experimental plan of the study was duly approved by the Animal Ethics Committee G. B. Pant University of Agriculture and Technology (GBPUAT), Pantnagar, Uttarakhand, India.

### Resource population

The study was conducted on 10 adults apparently healthy Uttara fowl of either sex reared at instructional poultry farm, College of Veterinary and Animal Sciences, GBPUAT, Pantnagar.

### Sample processing

#### TEM

The blood samples from the bird were collected in 5 ml syringe using ethylenediamine tetraacetic acid (EDTA) as anticoagulant, transferred into siliconized centrifuge tubes and spun at 3000 rpm for 45 min. The plasma was drained off and the buffy coat was fixed for 5 h at 5°C in the standard fixative (5% glutaraldehyde in 0.1 M phosphate buffer, pH 7.4). The fixative was gently removed. Fragile buffy coat disc was carefully removed with the help of a sharp pointed scalpel. After removal, the disc was put in a petridish containing phosphate buffered saline (pH 7.4). The disc was cut into several blocks of about 1-2 mm size. The blocks were washed three times with 0.1 M phosphate buffer (pH 7.4) for 15 min each. The blocks were again fixed at room temperature for 1 h using 1% osmium tetraoxide.

The blocks were dehydrated using graded alcohol. They were then placed into two changes of 100% toluene for 5 min each, before being transferred to a mixture of equal parts of araldite and toluene for overnight at room temperature. Impregnation was carried out in the fresh changes of araldite and continued for 2 days at room temperature. The blocks were finally embedded in another change of fresh araldite and polymerized for 3 days at room temperature.

The thin sections (60-70 nm) were cut on an ultramicrotome and placed on copper grids. The sections were stained in a saturated solution of uranyl acetate in 50% alcohol for 15 min, followed by lead citrate for 15 min and examined under TEM (JEOL JEM-1011) operated at 60-80 KV at TEM laboratory facility at GBPUAT, Pantnagar.

### SEM

Samples of venous blood from the birds were collected from the wing vein in 5 ml syringe using EDTA as anticoagulant. Few drops of blood were transferred into siliconized centrifuge tubes, equal amount of 5% glutaraldehyde was added and the blood was fixed for 1 h. The mix was spun at 3000 rpm for 30 min. The plasma was decanted and buffy coat was taken out in another tube. The blood cells were washed three times with 0.1 M phosphate buffer solution (pH 7.4) by centrifuging at 2000 rpm for 2 min. These samples of fixed cells were processed in SEM laboratory facility, GBPUAT, Pantnagar. There the cells were resuspended in distilled water and repeated washings were performed. The film of the blood cells was made on a clean circular glass coverslip. The blood film was coated with gold sputter coating using JFC-1600 Auto Fine Coater and observed under JEOL JSM-6610 LV SEM.

## Results

### TEM

#### Heterophils

**Figure-1:**
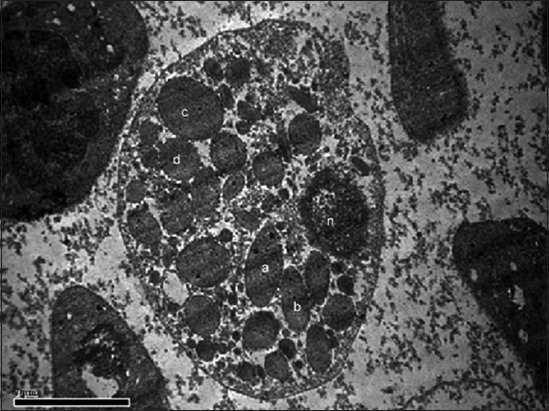
Transmission electron photomicrograph showing heterophil with large electron dense elliptical granule (a), medium electron dense oval granule (b), large electron dense round granule (c), medium electron dense round granule (d) and nucleus (n). Uranyl acetate and lead citrate ×19,900.

**Figure-2:**
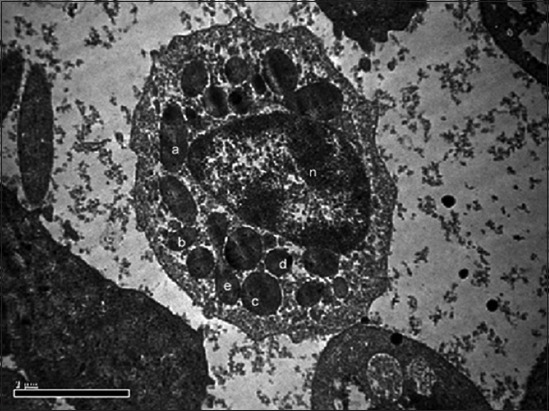
Transmission electron photomicrograph showing heterophil with large electron dense elliptical granule (a), medium electron dense oval granule (b), large electron dense round granule (c), medium electron dense round granule (d), dumbbell-shaped granule (e) and nucleus (n). Uranyl acetate and lead citrate ×19,900.

Ultrastructurally, the heterophils were irregularly round in shape. The nucleus was bilobed or trilobed. The nuclear lobes contained more amount of heterochromatin than euchromatin. The euchromatin occupied a major central portion of the nucleus, whereas heterochromatin was distributed mainly toward periphery in the form of large and small patches (Figure-1). The cytoplasm was laden with membrane-bound cytoplasmic granules showing pleomorphism (Figure-2). The cytoplasmic granules varied greatly in shape, size, and density. The largest granules were electron dense and elliptical in shape, measuring 0.4-1.6 µm in length. The second types of granules were medium electron dense and oval in shape measuring 0.6-0.8 µm in length. The third types of granules were large electron dense and round in shape. Fourth types of granules were medium electron dense and round in shape. In few heterophils, electron dense dumbbell-shaped granule was also seen and that may be due to fusion of two oval granules. The Golgi apparatus, mitochondria, and endoplasmic reticulum were seen in the cytoplasm. Mitochondria were oval to round in shape. Few vacuoles were observed at periphery which contained phagocytized material inside.

#### Eosinophils

**Figure-3:**
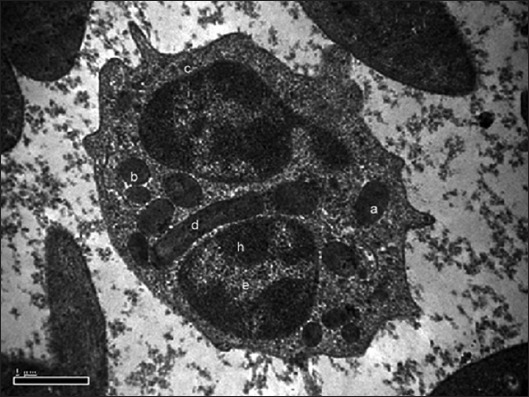
Transmission electron photomicrograph showing eosinophil with large electron dense elliptical granule (a), medium electron dense round granule (b), small electron dense round granule (c), long electron dense rod-shaped granule (d), euchromatin (e) and heterochromatin (h). Uranyl acetate and lead citrate ×26,500.

**Figure-4:**
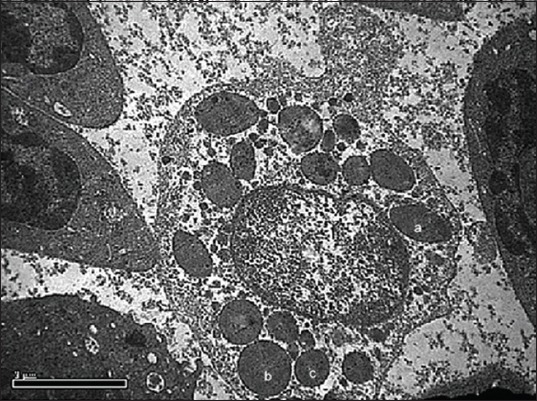
Transmission electron photomicrograph showing eosinophil with large electron dense elliptical granule (a), large electron dense round granule (b), medium electron dense round granule (c), small electron dense round-shaped granule (d) and nucleus (n). Uranyl acetate and lead citrate ×19,900.

The eosinophils under TEM were irregularly circular in outline showing pseudopodia and finger-like cytoplasmic processes. The nucleus was usually bilobed (Figure-3). There was a distinct nuclear membrane. The heterochromatin was concentrated toward the periphery in the form of large and small patches. The euchromatin was centralized. The cytoplasmic granules were distributed throughout the cytoplasm and varied greatly in shape and size. The first types of granules were electron dense and elliptical in shape. The second types of granules were large electron dense and round in shape. The third types of granules were medium electron dense and round in shape. The fourth types of granules were small electron dense and round in appearance (Figure-4). In few eosinophils, long rod-shaped electron dense granule was visible.

#### Basophils

**Figure-5:**
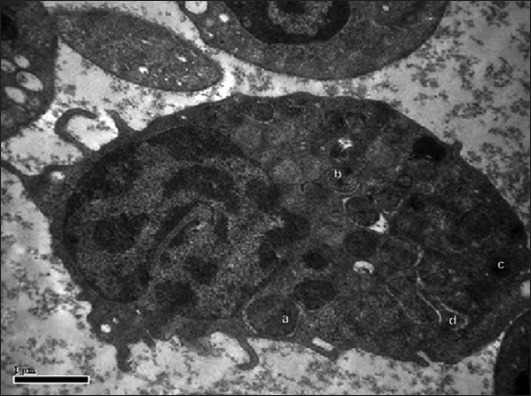
Transmission electron photomicrograph of basophil showing large electron dense round granule (a), round honeycomb-like granule (b), small electron dense round granule (c), and cytoplasmic process (cp). Uranyl acetate and lead citrate ×26,500.

**Figure-6:**
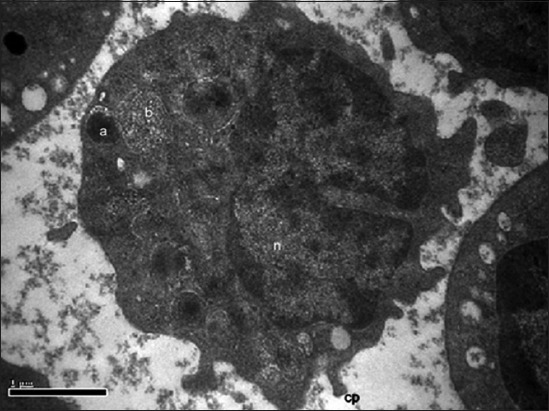
Transmission electron photomicrograph of basophil showing electron-dense round granule (a), electron lucent round honeycomb-like granule (b), cytoplasmic process (cp) and nucleus (n). Uranyl acetate and lead citrate ×33,200.

The basophils were irregularly circular in outline containing small hook-like cytoplasmic processes (Figure-5). The majority of basophils contained a single, non-lobulated, eccentrically placed, indented nucleus. The nuclear membrane was distinct. The heterochromatin was distributed toward the periphery as well as in the center in the form of small and large patches. The cytoplasm was laden with cytoplasmic granules showing pleomorphism. Most of the cytoplasmic granules were electron dense and round in shape. Few granules were electron lucent showing a loose honeycomb-like stippling in the interior (Figure-6). The other cell organelles observed in the cytoplasm were mitochondria, Golgi apparatus, vesicles, and smooth as well as rough endoplasmic reticulum. Few small vacuoles were also seen toward the periphery of the cytoplasm. Few loop-like cytoplasmic processes were observed toward the nuclear end of the cell.

### SEM

**Figure-7:**
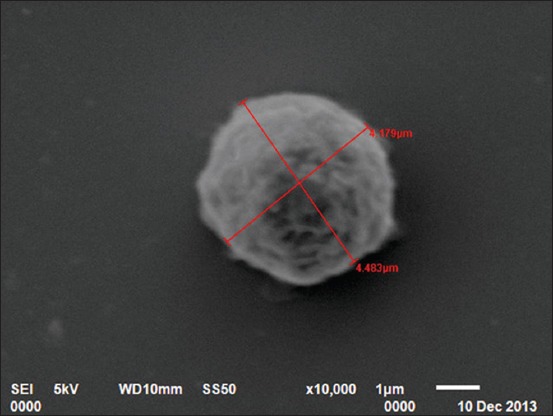
Scanning electron photomicrograph of blood cells showing small leukocyte with mushroom-like appearance ×10,000.

**Figure-8:**
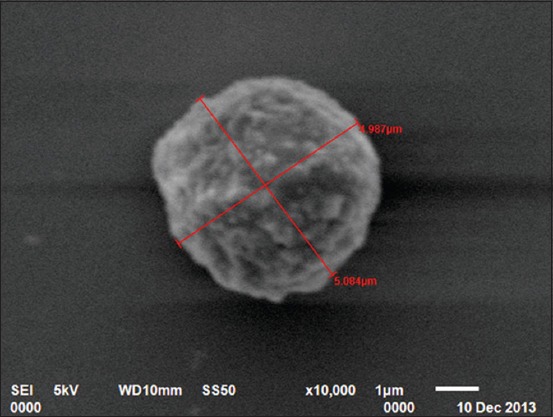
Scanning electron photomicrograph of blood cells showing medium leukocyte with mulberry-like appearance ×10,000.

**Figure-9:**
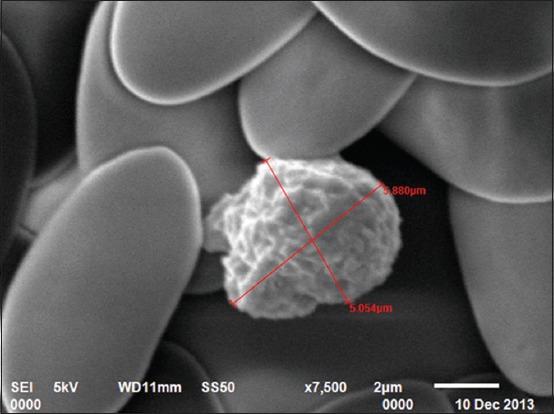
Scanning electron photomicrograph of blood cells showing large leukocyte with cauliflower-like appearance ×7500.

**Figure-10:**
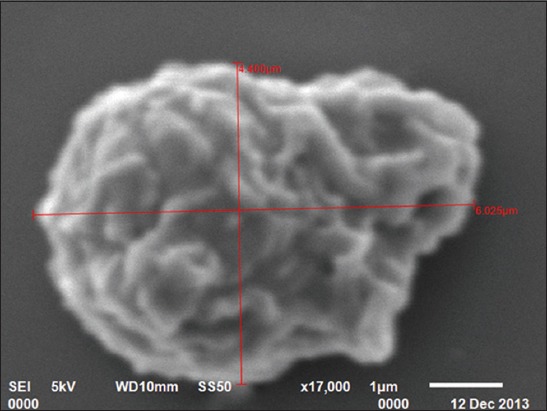
Scanning electron photomicrograph of blood cells showing large leukocyte with membrane-like ruffles ×17,000.

In the present study, four types of leukocytes were observed under SEM. First types of leukocytes were small in size and showed blunt, irregularly round mushroom type outgrowth on their surface. These types of leukocytes also had a varying number of microvilli on their surface (Figure-7). The second types of leukocytes were medium in size having irregularly round mulberry-like surface outgrowths (Figure-8). The third types of leukocytes were large in size and showed cauliflower-like appearance. Their cell surface showed narrow ridge-like profiles and small ruffles (Figure-9). The fourth types of leukocytes were also large with more prominent, membrane-like ruffles (Figure-10).

## Discussion

The nucleus of the heterophil in the Uttar fowl was multilobulated in appearance. The euchromatin was distributed in the central portion of the nucleus and heterochromatin toward periphery in the form of patches. Heterophils with lobulated nucleus with heterochromatin distributed in the periphery were reported in ostrich [5]. Lobulated nuclei with 2-5 lobes [6] were reported in pig neutrophils in which the heterochromatin occupied a major peripheral portion of the nuclear lobes, whereas the euchromatin was comparatively less and centrally located in the form of patches. The cytoplasmic granules of pleomorphic nature in the Uttara fowl were in collaboration with the findings in painted storks [7] in which numerous membrane-bound pleomorphic granules were seen in the cytoplasm of the heterophil. In place of four types of granules observed in Uttara fowl heterophil, three types of granules were reported in chicken [8], pigeon [9], and aves [10]. There were only two types of granules reported in ostrich heterophil [5]. Type I granules were fusiform with different size and Type II were smaller with varied electron density. Reports also suggested [11] large elliptical-shaped electron dense cytoplasmic granules in Kadaknath fowl. Slightly different findings of granules in avian heterophils were reported [12]. Their primary granules appeared electron-dense fusiform rods (1.5 µm × 0.5 µm), secondary granules (diameter, 0.5 µm) were less dense containing eccentric inclusions, and tertiary granules (0.1 µm) had a dense core that was separated from a membranous envelope of an electron-lucent area. However, the primary granules were almost similar in size to elliptical-shaped granules of the present study. In few heterophils, electron dense dumbbell-shaped granule was also seen, that may be due to fusion of two oval granules. The Golgi apparatus, mitochondria, and endoplasmic reticulum were seen in the cytoplasm. Mitochondria were oval to round in shape. Few vacuoles were observed at periphery which contained phagocytized material inside. However, in Kadaknath fowl [11], vesicles were observed.

The eosinophils contained usually bilobed nucleus with central euchromatin and peripheral heterochromatin arrangement. Similar observations of the bilobed nucleus with similar chromatin pattern [11] were reported in eosinophil of Kadaknath fowl. The eosinophils contained lobed nuclei in painted storks [7]. The eosinophils appeared round to oval shaped with long and narrow cytoplasmic processes in the horse [13]. The cytoplasmic granules were distributed throughout the cytoplasm and were pleomorphic in appearance in the Uttara fowl. These findings were somewhat similar to the findings of duck eosinophil in which round, oval, or elongated granules were observed but the findings were slightly different from goose (oval and round granules), quail (oval and round granules), fantail and homing pigeon (dense large round, less dense round and round granule with empty area within), and turkey (large irregular-shaped granules) eosinophils [14]. The round-shaped eosinophilic granule characteristics were similar as in guinea fowl [15] and in painted storks [7]. Rod-shaped granules along with spherical granules were also observed in birds [10]. The reports suggested that granules in fowl eosinophils were round to oval in shape, but some granules had appearance of vacuoles [16].

The majority of basophils contained a single, non-lobulated, eccentrically placed, indented nucleus. Similarly, the basophils with single non-lobulated nucleus were found in the duck, goose, and turkey [9]. Most of the basophilic cytoplasmic granules of Uttara fowl were electron dense and round in shape. However, few granules were electron lucent showing a loose honeycomb-like stippling in the interior. The basophils contained four types of double membrane-bound cytoplasmic granules in guinea fowl [15]. Electron dense oval or elongated granules were reported in the Kadaknath fowl [11]. The membrane-bound pleomorphic cytoplasmic granules were reported in painted stork [7] and sheep [17]. In pig basophils, the cytoplasmic granules were comparatively less when compared with neutrophils and eosinophils [6]. The cell organelles observed under TEM in the cytoplasm of basophil in the Uttara fowl were mitochondria, Golgi apparatus, vesicles, and smooth as well as rough endoplasmic reticulum. The cell organelles such as mitochondria, very small empty vesicles, scarce, and poorly developed endoplasmic reticulum were observed in the basophil of domestic fowl [16]. Reports suggest the presence of mitochondria and ribosomes in painted stork [7].

In the present study, four types of leukocytes were observed under SEM. These types of leukocytes had varying number of microvilli on their surface. The leukocytes with microvillus on the surface were also reported [18] in guinea fowl. Four types of leukocytes were also observed in Kadaknath fowl [19] but with different surface morphology as compared to present findings of Uttara fowl. In our findings, cells had different cellular processes such as blunt, irregularly round, mushroom type; mulberry type; cauliflower type, and membrane-ruffled type. However, in Kadaknath fowl, the leukocytes with only coarse wafer-like processes and mushroom-like processes on the surface were observed. In the present study, the leukocytes cannot be conclusively divided into granulocytes and agranulocytes. However in chicken [20], the cells, with ridge-like profiles having membrane ruffles, were considered as polymorphonuclear leukocytes and cells with more prominent membrane ruffles as monocytes.

## Conclusions

Ultrastructurally, the heterophils were irregularly round in shape. The nucleus was bilobed or trilobed. The cytoplasm of heterophil was laden with pleomorphic membrane-bound granules. The cytoplasmic granules varied greatly in size and shape, *viz*., large elliptical-, medium oval-, large round-, and medium round-shaped granules. The eosinophils under TEM were irregularly circular in outline showing pseudopodia and finger-like cytoplasmic processes. The cytoplasmic granules of eosinophils showed pleomorphism with elliptical-, round-, and rod-shaped granules. The basophils were irregularly circular in outline containing small hook-like cytoplasmic processes. The majority of basophils contained a single, non-lobulated, eccentrically placed, indented nucleus. Most of the cytoplasmic granules were electron dense and round in shape. Few granules were electron lucent showing a loose honeycomb-like stippling in the interior. The SEM revealed four different types of leukocytes in the blood.

## Authors’ Contributions

MKI carried out this study and wrote this manuscript. MM planned, guided, and helped in interpreting and finalizing the results in this research work. BS helped in the processing of samples in this study. IS guided in the interpretation of electron microscopic photomicrographs. All authors have read and approved the final version of the manuscript.
